# Aminoglycoside Allergic Reactions

**DOI:** 10.3390/pharmacy7030124

**Published:** 2019-08-29

**Authors:** Lindsey M. Childs-Kean, Kristy M. Shaeer, Sheeba Varghese Gupta, Jonathan C. Cho

**Affiliations:** 1 Department of Pharmacotherapy and Translational Research, University of Florida College of Pharmacy, Gainesville, FL 32610, USA; 2 Department of Pharmacotherapeutics and Clinical Research, University of South Florida College of Pharmacy, Tampa, FL 33612, USA; 3 Department of Pharmaceutical Sciences, University of South Florida College of Pharmacy, Tampa, FL 33612, USA; 4 Department of Clinical Sciences, Ben and Maytee Fisch College of Pharmacy, The University of Texas at Tyler, Tyler, TX 75799, USA

**Keywords:** aminoglycosides, allergy, hypersensitivity, gentamicin, tobramycin, amikacin

## Abstract

Aminoglycosides are antimicrobial agents that are primarily used for infections caused by Gram-negative pathogens. The purpose of this article is to review the allergic reactions reported in the published literature to aminoglycoside antibiotics. A thorough PubMed search was conducted and excluded non-allergic adverse reactions to aminoglycosides. Allergic reactions to aminoglycosides occur infrequently, but can include cutaneous reactions as well as systemic reactions, including anaphylaxis. Of the evaluated aminoglycosides, gentamicin had the most reported allergic reactions, including the most reports of anaphylaxis, followed by tobramycin, and then amikacin. Most reports of allergic reactions occurred in patients who had a prior exposure to some dosage form of an aminoglycoside. Cross-reactivity among aminoglycosides is common and occurs due to the similarities in their chemical structures. Desensitization protocols to tobramycin have been described in the literature.

## 1. Introduction

Antibiotics are one of the most common causes of life-threatening medication allergies [1]. These allergic reactions can be broadly categorized into immediate (IgE-mediated) or non-immediate reactions (T cell-mediated). Patients with immediate allergic reactions can present clinical manifestations that include urticaria, angioedema, and anaphylaxis, whereas those with non-immediate reactions can present symptoms ranging from contact dermatitis to a maculopapular rash [1]. A vast number of hypersensitivity reactions have been reported from the beta-lactam class (up to 15% of patients), but allergic reactions from aminoglycosides are less frequently reported (<2%) [1,2]. Similar to beta-lactam antibiotics, non-immediate reactions to aminoglycosides are more commonly seen [2]. Contact dermatitis from topical aminoglycoside use is the most frequently reported reaction with aminoglycosides, even when compared with other topical medications [3,4]. Due to the infrequent nature of aminoglycoside allergies, a review of the current literature on aminoglycoside hypersensitivity reactions and associated management is lacking. This paper will review the medicinal chemistry, indications, reported allergic reactions, and desensitization protocols associated with aminoglycoside utilization.

## 2. Medicinal Chemistry

### 2.1. Aminoglycoside Structure

**Figure 1:**
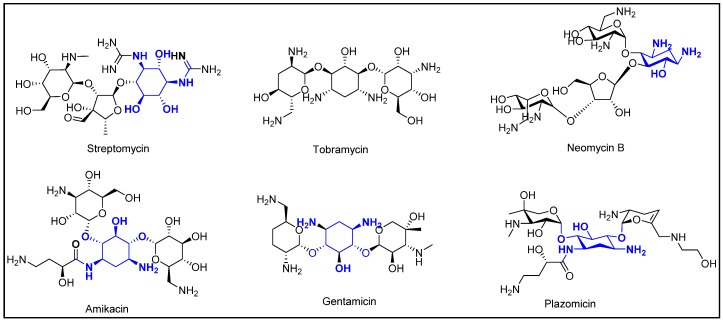
Chemical structures of aminoglycosides, with structural differences highlighted in blue [5].

The structure of aminoglycosides consists of a hexose ring, to which various amino sugars are attached via glycosidic linkages. Aminoglycosides can be classified into two main structural classes based on the aminocylitol nucleus: streptidine (streptomycin) and deoxystrepatamine (gentamicin, tobramycin, amikacin, kanamycin, neomycin, and plazomicin) (Figure 1). Irrespective of their structural differences, all aminoglycosides exhibit concentration-dependent bactericidal activity through inhibition of protein synthesis. The structural difference seems to play an important role in escaping the bacterial resistance mechanisms, especially by offering structural robustness against metabolizing enzymes, such as Aminoglycoside Modifying Enzymes (AMEs), and target-modifying 16S rRNA methyl transferases (16S-RMTases), produced by the bacteria [5]. Amikacin and plazomicin have been shown to have increased stability against AMEs compared to gentamicin [5]. All currently marketed aminoglycosides are affected by 16S-RMTases, rendering them inactive against the organisms producing the enzyme [5].

### 2.2. Structural Aspects behind Cross-Reactivity

**Figure 2:**
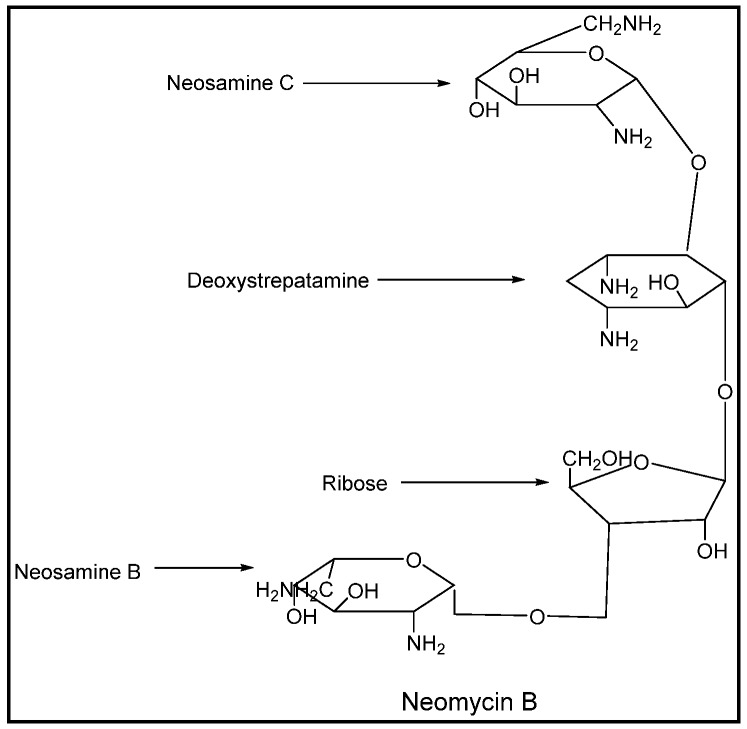
Structural constituents of Neomycin B [5].

Neomycin has antigenic properties possessing diamino sugars called neosamines in its structure. Similar diamino sugars with minor modifications are present in kanamycin and tobramycin. As a result of the structural similarity between tobramycin and neomycin, up to 65% of patients allergic to neomycin are shown to have a cross-allergic reaction to tobramycin [6]. Figure 2 shows the structural constituents of neomycin B. Kanamycin has also been shown to have cross-sensitivity with neomycin, owing to the presence of neosamine-like groups in its structure [7]. Gentamicin has also shown cross-reactivity with neomycin. In the case of gentamicin, the antigenicity is contributed by the deoxystreptamine group in its structure, which is also present in neomycin [8]. Generally speaking, cross-reactivity among aminoglycosides with the deoxystreptamine group (gentamicin, tobramycin, amikacin, kanamycin, neomycin, and plazomicin) is at least 50% [9]. For this reason, all deoxystreptamine-containing aminoglycosides carry a contraindication against use if a patient has a known hypersensitivity to another deoxystreptamine-containing aminoglycoside [10,11,12,13].

As mentioned above, cross-reactivity is due to specific functional groups present in these antibiotics. Cross-reactivity between the deoxystreptamine-containing aminoglycosides and streptidine-containing streptomycin has not been observed. Streptidine in streptomycin is responsible for its antigenicity; therefore, deoxystreptamine-sensitive individuals are less likely to develop allergic cross-reactions to streptomycin therapy [14].

## 3. Medical Conditions for Which Aminoglycosides Are Used

Aminoglycosides are useful in the treatment of a wide variety of diseases, primarily infections caused by Gram-negative aerobic bacilli, typically in combination with another antimicrobial or as monotherapy for urinary tract infections (Table 1) [10,11,12,15,16,17]. Aminoglycoside monotherapy has been associated with a significantly higher rate of bacteriological failure at the end of therapy in the treatment of nonurinary sources (e.g., pneumonia, abscess, central nervous system, etc.) [18,19].

Gentamicin has activity against many Gram-negative organisms such as all Enterobacteriaceae spp. (except Providencia stuartii), Pseudomonas aeruginosa, Aeromonas spp., Haemophilus influenzae, Brucella spp., Moraxella spp., Pasturella multocida, and Francisella tularensis, and occasionally Acinetobacter baumannii [11,16]. Gentamicin and tobramycin can be given in combination with other cell wall-active antimicrobials in order to capitalize on their synergistic action against Gram-positive organisms [16,17]; gentamicin has synergistic activity in combination with beta-lactams or vancomycin against several Gram-positive organisms (e.g., Bacillus spp., Enterococcus faecalis, Listeria monocytogenes, Staphylococcus aureus, Streptococcus spp.) [16]. Compared to gentamicin, tobramycin has similar activity against many Gram-negative organisms, such as all Enterobacteriaceae spp. (except Providencia stuartii), and Haemophilus influenzae; however, it has higher potency against Pseudomonas aeruginosa and Acinetobacter baumannii [12,17,20,21,22]. Tobramycin has little activity against Gram-positive organisms, but may be given in combination with cell wall-active agents for treatment of E. faecalis-associated infections [17]. Compared to tobramycin, amikacin has activity against all Enterobacteriaceae spp., H. influenzae, P. multocida, and higher potency against Pseudomonas aeruginosa and Acinetobacter baumannii [10,15]. Amikacin may have activity against many Mycobacterium spp., Staphylococci spp., and Nocardia asteroids [15]. Plazomicin, the newest aminoglycoside, has activity against extended-spectrum beta-lactamase-producing and carbapenem-resistant Enterobacteriaceae spp., and is reserved for patients with limited or no other treatment options [13,23].

In addition to parenteral administration for systemic infections, most aminoglycosides come in other dosage forms that are used clinically. For example, gentamicin and tobramycin are available in topical dosage forms to treat ophthalmic, otic, and skin infections [21,22,24,25,26]. They are also included in bone cement and beads to prevent and treat bone and joint infections [27]. Tobramycin and amikacin are available in inhaled dosage forms, such as a nebulizer solution and an inhalation powder or suspension, to treat pulmonary infections [20,28].

If the patient has an IgE-mediated aminoglycoside allergy or fails or refuses drug desensitization (described later), then a provider should select empiric or targeted alternative therapies deemed appropriate for the type and cause of the infection. The most likely alternative would be another broad-spectrum agent (e.g., beta-lactam/beta-lactamase inhibitor, fourth generation cephalosporin, carbapenem, tetracycline, or macrolide).

## 4. Documented Allergic Reactions

Overall, allergic reactions to aminoglycosides reported in the literature are rare. Aminoglycosides come in multiple dosage forms, including topical, parenteral, and inhaled, and there have been reports of reactions from each dosage form, some being localized reactions, and others being systemic reactions [30,31,32,33,34,35,36,37,38,39,40,41,42,43,44,45,46,47,48,49,50,51,52,53,54,55,56,57,58,59]. Below and in Table 2, Table 3 and Table 4 is a summary of the reactions caused by gentamicin, tobramycin, and amikacin. No allergic reactions to the newest aminoglycoside, plazomicin, were found in the literature. Most reported reactions occurred in children or patients over the age of 55 years, and most were confirmed via a patch test or a rechallenge. Additionally, many of the reactions involved a patient who had received a prior aminoglycoside, usually as a topical or ophthalmic dosage form. Most patients described were switched to an alternative antibiotic with resolution of symptoms; other patients underwent desensitization to the aminoglycoside.

The most frequently reported type of allergic reaction for gentamicin, tobramycin, and amikacin was cutaneous, and the reactions were caused by a variety of dosage forms (Table 2). For gentamicin, there were reports of allergic dermatitis, contact sensitivity, ulcerative dermatitis, urticaria, rash, and exfoliative erythroderma [30,31,32,33,34,35,36,37,38,39,40]. Reported cutaneous reactions with tobramycin included contact dermatitis, exfoliative dermatitis, rash, urticaria, toxic epidermal necrosis (given concomitantly with meropenem), Drug Rash with Eosinophilia and Systemic Symptoms (DRESS) syndrome (given concomitantly with piperacillin/tazobactam), and fixed exanthema [40,41,42,43,44,45,46,47,48]. The only skin-related reaction to amikacin reported was DRESS [49].

There are reports of anaphylaxis with both gentamicin and amikacin, and all of these cases result from parenteral use of the drug (Table 3) [50,51,52,53,54,55]. Two of the case reports involving gentamicin identified that the patient had previous been treated with at least one aminoglycoside, which could have caused the sensitization. Interestingly, both of these reports implicated either a topical dosage form or the use of gentamicin in bone cement, not a previous parenteral administration of an aminoglycoside, as the cause of sensitization [51,54].

Table 4 summarizes other allergic reactions that have been reported to aminoglycosides [47,56,57,58,59]. Bronchospasms with or without other signs and symptoms (eosinophilia, urticaria, pruritus) have been reported with use of tobramycin. One case report described a patient who experienced bronchospasm with eosinophilia, with inhaled tobramycin [58]. Another case report described a patient with bronchospasm, urticaria, and pruritus after receiving intravenous tobramycin [59]. The case report describing bronchospasm without any other signs and symptoms occurred after ocular administration of tobramycin [57]. Other reported reactions with tobramycin included arthralgia and fever [47]. A reported reaction with questionable attribution to gentamicin (due to concomitant administration with penicillin) was serum sickness [56].

## 5. Desensitization

Antimicrobial desensitization procedures have been conducted since the 1940s and are more commonly completed in patients with allergic reactions to penicillin antibiotics [4]. Desensitization typically requires several hours to complete, and can be accomplished through administration of incremental doses of an antimicrobial agent, resulting in immune tolerance to that antimicrobial. Due to the infrequent nature of aminoglycoside allergies, desensitization procedures are not generally performed, but limited data, restricted to case reports and case series, have demonstrated success when utilized [38,40,47,59].

Four articles were identified describing desensitization protocols and outcomes in patients with aminoglycoside allergies [38,40,47,59]. All desensitization procedures were conducted in patients with cystic fibrosis requiring tobramycin administration. Earl et al. described a tobramycin desensitization due to a case of urticarial reactions to both beta-lactam and aminoglycoside antibiotics in a 15-year-old patient receiving treatment for a left upper-lobe lung abscess [40]. The patient had a history of pulmonary infection due to *Pseudomonas* sp. and experienced generalized urticaria to both intravenous and inhaled tobramycin therapy. Prior to desensitization, the patient received pretreatment with theophylline and inhaled isoproterenol to suppress any pulmonary responses. Antihistamines were not administered due to the risk of masking early cutaneous reactions from tobramycin. The initial dose of tobramycin during the desensitization protocol was 1 mcg diluted in 20 mL of normal saline, and given intravenously over 20 min. Ten minutes after the end of the infusion, the patient received double the amount of tobramycin as provided in the previous dose. This procedure was repeated 17 times over eight hours until a cumulative dose of 80 mg of tobramycin was given. No reactions were noted, with the exception of a transient rash after doses 9 (0.256 mg) and 10 (0.512 mg) of tobramycin. The patient ultimately received 80 mg of intravenous tobramycin every six hours and did not experience any allergic reactions for the remainder of treatment [40].

The protocol by Earl et al. was also used by Schretlen-Doherty et al. in an 18-year-old male for treatment of a cystic fibrosis exacerbation [59]. This patient developed shaking, rash, and pruritus secondary to intravenous tobramycin administration. After desensitization, no allergic reactions were noted for the remainder of the 14-day course [59].

Similar success was found in a retrospective review of patients that underwent tobramycin desensitization [47]. Seven out of eight (88%) patients successfully completed tobramycin desensitization; one person felt unwell and experienced fever during desensitization and was unable to complete the full desensitization protocol. This desensitization protocol differed from the protocol discussed previously; this protocol utilized a seven-step approach over 140 min, with 10-fold increases in tobramycin doses. Additionally, oral antihistamines and steroids were provided as pretreatment at the discretion of the provider [47].

Spigarelli et al. described their experience with an inhaled tobramycin desensitization protocol in a 9-year-old cystic fibrosis patient with pulmonary exacerbation [38]. The patient had a history of rash when administered intravenous gentamicin, and developed a rash during inhaled tobramycin therapy. As part of the desensitization protocol, the patient underwent 15 treatments every 2 h via nebulizer. The initial dose was 0.3 mg of tobramycin in 5 mL of normal saline; subsequent doses were gradually increased until the full dose of 300 mg was administered [38]. No adverse reactions were noted during the desensitization protocol and pulmonary function tests were not performed as respiratory symptoms were not exhibited by the patient. After desensitization was completed, the patient received nine months of tobramycin inhalation therapy without any complications or signs of rash or fever [38].

## 6. Conclusions

Allergic reactions due to aminoglycoside use are infrequently reported, with non-immediate, cutaneous reactions being the most commonly reported. Strategies to overcome hypersensitivity reactions, such as desensitization, have been utilized with successful outcomes. It is prudent to change to an alternative antibiotic if the patient experiences an immediate hypersensitivity reaction (e.g., anaphylaxis, hives), and changing antibiotics can be considered for other reactions, such as rash.

Currently available aminoglycosides are similar in structure and are likely to cause cross-reactivity. For this reason, if a patient has a hypersensitivity reaction to one aminoglycoside, the patient should not receive another aminoglycoside, unless a desensitization protocol has been instituted. Despite the small risk of allergic reactions to aminoglycosides, they still have clinical utility for a number of infectious conditions.

## Figures and Tables

**Table 1:** Summary of FDA approved and unapproved clinical uses of aminoglycosides.

Clinical Uses	Aminoglycosides Indicated	Comments
**FDA Approved Uses**		
Bacterial meningitis and CNS [10,11,12,29]	AMK, GEN, TOB	
Bacterial sepsis [10,11,12,29]	AMK, GEN, TOB	
Bone and joint [10,11,12,29]	AMK, GEN, TOB	
Burn [11,29]	AMK	
Cystic fibrosis-associated respiratory tract infection [10,12,20,29]	AMK, TOB	
Intra-abdominal including peritonitis [10,11,12,20,29]	AMK, GEN, TOB	In combination with agent with Gram-positive and anaerobic activity
Ocular [21,22,24,25,29]	GEN, TOB	
Otic (external) [26]	GEN	
Refractory MAC lung disease [28]	ALIS	In combination antibacterial drug regimen with limited or no alternative treatment options
Lower respiratory tract (severe) [10,11,12,29]	AMK, GEN, TOB	In combination with beta-lactam, beta-lactam/beta-lactamase inhibitor, or 3rd or 4th generation cephalosporin
Skin and soft tissue [10,11,12,29]	AMK, GEN,	
Urinary tract (severe, complicated) [10,11,12,13,29]	AMK, GEN, TOB, PLZ	
Post-operative [10]	AMK	
**FDA Unapproved Indications**		
Actinomycosis [15]	AMK	
Bacterial endocarditis [29]	AMK, GEN	In combination with beta-lactam or vancomycin
Brucellosis [16]	GEN	
Decontamination of GIT [16]	GEN	
Febrile neutropenia (adjunct) [29]	AMK, GEN, TOB	In combination therapy
Female genital infection [29]	GEN	
Impregnated cement and beads [27]	GEN, TOB	
Mycobacteriosis [29]	AMK	In combination therapy
Ménière’s disease [16,29]	GEN	
Necrotizing enterocolitis in fetus or newborn [29]	GEN	
Nocardiosis [29]	AMK	
Pelvic inflammatory disease (severe) [29]	GEN	In combination with clindamycin
Plague [16,29]	GEN	
Surgical prophylaxis [16,29]	GEN, TOB	
Tularemia [16,29]	GEN	
Uterus (peripartum, postnatal) [29]	GEN	

Abbreviations: ALIS = amikacin liposome inhalation suspension; AMK = amikacin; CNS = central nervous system; FDA = Food and Drug Administration; GEN = gentamicin; GIT = gastrointestinal tract; MAC = *Mycobacterium avium* complex; PLZ = plazomicin; TOB = tobramycin.

**Table 2:** Cutaneous reactions to aminoglycosides.

Aminoglycoside Implicated	Reaction	Patient Demographics	Dosage Form	Concomitant Drugs	Patch Test or Rechallenge Confirm Aminoglycoside Allergy?	Sensitization to Previous Dosage Form of Aminoglycoside?
Gentamicin	Allergic Dermatitis [30]	84-year-old female	Intravenous	None	Yes	Yes, topical
	Allergic Dermatitis [31]	74-year-old female	Bone cement	None	Yes	Yes, ongoing bone cement
	Allergic Dermatitis [32]	30-year-old female	Intravenous	Ampicillin, bupivacaine, lidocaine, ketorolac, pethidine, metamizole magnesium	Yes	Yes, topical
	Allergic Dermatitis [33]	55-year-old female	Ophthalmic solution	None	Yes	Yes, ophthalmic
	Allergic Dermatitis [34]	79-year-old	Intra-articular	None	Yes	Yes, ophthalmic
	Contact Dermatitis [35]	5 newborns	Ophthalmic ointment	Unknown	Unknown	Unknown
	Contact Sensitivity [36]	50-year-old male	Ophthalmic solution	None	Yes	Yes, ophthalmic
	Exfoliative Erythroderma [37]	66-year-old male	Intravenous	Ceftozoxime	Yes	Yes, otic
	Rash [38]	9-year-old male	Intravenous	Piperacillin/tazobactam	Unknown	Unknown
	Ulcerative Dermatitis [39]	26 newborns	Ophthalmic ointment	Unknown	Unknown	Unknown
Gentamicin and Tobramycin, different episodes	Urticaria [40]	15-year-old female	Intravenous	None	Yes	Unknown
Tobramycin	Allergic Contact Dermatitis [41]	32-year-old female	Otic drops	Betamethasone, sulfamethazine	Yes	Unknown
	Conjunctivitis [42]	59-year-old female	Ophthalmic suspension	Dexamethasone, atropine, timolol, brominidine, lanatoprost, brinzolamide	Yes	Unknown
	Conjunctivitis [43]	70-year-old female	Ophthalmic ointment	Loxacin, homatropine hydrobromide, tropicamide, phenylephrine, diclofenac	Yes	Unknown
	Drug Rash with Eosinophilia and Systemic Symptoms (DRESS) [44]	4-year-old female	Intravenous	Piperacillin/tazobactam	Unknown	Unknown
	Erythroderma, Exfoliative dermatitis [45]	55-year-old woman	Intravenous, Intraperitoneal	Vancomcyin, clindamycin	Yes	Unknown
	Fixed exanthema [46]	69-year-old female	Intramuscular	None	No	Yes, ophthalmic
	Rash [38]	9-year-old male	Inhaled	Pancre-lipase, ADEK vitamins, rh DNAse, albuterol	Yes	Yes, Intravenous
	Rash [47]	3 patients	Unknown	Unknown	Unknown	Unknown
	Toxic epidermal necrosis [48]	42-year-old male	Intravenous	Meropenem, vancomycin, oseltamivir, ciprofloxacin	Unknown	Unknown
	Urticaria [47]	3 patients	Unknown	Unknown	Unknown	Unknown
Amikacin	DRESS [49]	42-year-old male	Intravenous	Clindamycin, vancomycin	Yes	Unknown

**Table 3:** Anaphylactic reactions to aminoglycosides.

Aminoglycoside Implicated	Reaction	Patient Demographics	Dosage Form	Concomitant Drugs	Patch Test or Rechallenge Confirm Aminoglycoside Allergy?	Sensitization to Previous Dosage Form of Aminoglycoside?
Gentamicin	Anaphylaxis [50]	66-year-old female	Intravenous	None	Unknown	Unknown
	Anaphylaxis [51]	70-year-old female	Intravenous	Cefotiam	Yes	Yes, topical
	Anaphylaxis, Urticaria [52]	69-year-old male	Intravenous	Midazolam	Yes	Unknown
	Anaphylaxis [53]	53-year-old female	Intramuscular	None	Yes	Unknown
	Anaphylaxis [54]	66-year-old female	Intravenous	Chlorhexidine, bupivacaine, dexamethasone, remifentanil, cisatracurium, suxamethonium, propofol	Yes	Yes, bone cement
Amikacin	Anaphylaxis [55]	Newborn male	Intravenous	None	Unknown	Unknown

**Table 4:** Other reactions to aminoglycosides.

Aminoglycoside Implicated	Reaction	Patient Demographics	Dosage Form	Concomitant Drugs	Patch Test or Rechallenge Confirm Aminoglycoside Allergy?	Sensitization to Previous Dosage Form of Aminoglycoside?
Gentamicin	Serum Sickness [56]	3-year-old male	Intravenous	Penicillin G, aspirin	Unknown	Unknown
Tobramycin	Arthralgia [47]	1 patient	Unknown	Unknown	Unknown	Unknown
	Bronchospasm [57]	79-year-old female	Ophthalmic ointment	Phenylephrine, prednisolone	Unknown	Unknown
	Bronchospasm, eosinophilia [58]	6-year-old male	Inhaled, Intravenous	Unknown	Unknown	Yes, Inhaled
	Bronchospasm, urticaria, pruritus [59]	18-year-old male	Intravenous	Albuterol, cromolyn, DNAase, pancreatic enzymes, phytonadione, multivitamins, ceftazidime	Unknown	Yes, Intravenous
	Fever [47]	1 patient	Unknown	Unknown	Unknown	Unknown
